# Primary pulmonary T-cell lymphoma after operation for small intestinal stromal tumor: A case report

**DOI:** 10.3389/fonc.2022.926121

**Published:** 2022-11-10

**Authors:** Zhiwei Peng, Li Yi, Yahong Tao, Zhiyong Chen, Ze Lin, Anjing He, Mengni Jin, Fanrong Liu, Minjing Zuo

**Affiliations:** ^1^ Department of Radiology, The Second Affiliated Hospital of Nanchang University, Nanchang, China; ^2^ Department of Pathology, The Second Affiliated Hospital of Nanchang University, Nanchang, China

**Keywords:** primary pulmonary T-cell lymphoma, primary pulmonary lymphoma, imatinib, gastrointestinal stromal tumors, case report

## Abstract

**Background:**

The risk of gastrointestinal stromal tumor (GIST) in combination with other primary malignancies is high, which occurs before and after the diagnosis of GIST. Primary pulmonary T-cell lymphoma is a rare type of non-Hodgkin lymphoma.

**Case presentation:**

We report a 53-year-old male patient who was admitted to our hospital with fever, cough, and expectoration for 2 weeks. Chest computed tomography (CT) showed a cavitary mass in the left lower lobe with multiple nodules in the upper lobes of both lungs. The patient had a history of surgery for small intestinal stromal tumors and was treated with oral imatinib after surgery. Lung biopsy was diagnosed as lymphomatoid granulomatosis, tending to grade 3. The pathological diagnosis was corrected by surgery and genetic testing for lung non-Hodgkin CD8-positive cytotoxic T-cell lymphoma with Epstein–Barr virus (EBV) infection in some cells. After multiple chemotherapies, the CT scan showed a better improvement than before. The patient is still under follow-up, and no tumor recurrence has been found.

**Conclusion:**

Patients with a history of GIST should be monitored for other malignancies. The clinical symptoms and imaging examinations of primary pulmonary T-cell lymphoma are not characteristic, and the definite diagnosis still depends on pathological examination. The patient was treated with the CHOP chemotherapy regimen after the operation, the curative effect was good.

## Introduction

Primary pulmonary lymphoma (PPL) refers to malignant lymphoma originating from the lymphoid tissue in the lung, originating from the bronchial mucosa-associated lymphoid tissue and/or the lymphoid tissue in the lung, and it only accounts for 0.5-1% of primary lung malignant tumors (1). The pathological types of PPL are divided into Hodgkin lymphoma (HL) and non-Hodgkin lymphoma (NHL). Low-grade B-cell lymphomas are mostly found in NHL, while T-cell lymphomas are rare (2). Other malignant tumors may occur before and after the diagnosis of GIST. We report a case of primary pulmonary T-cell lymphoma after surgery for a small intestinal stromal tumor.

## Case presentation

### Clinical diagnosis and treatment

**Figure 1:**
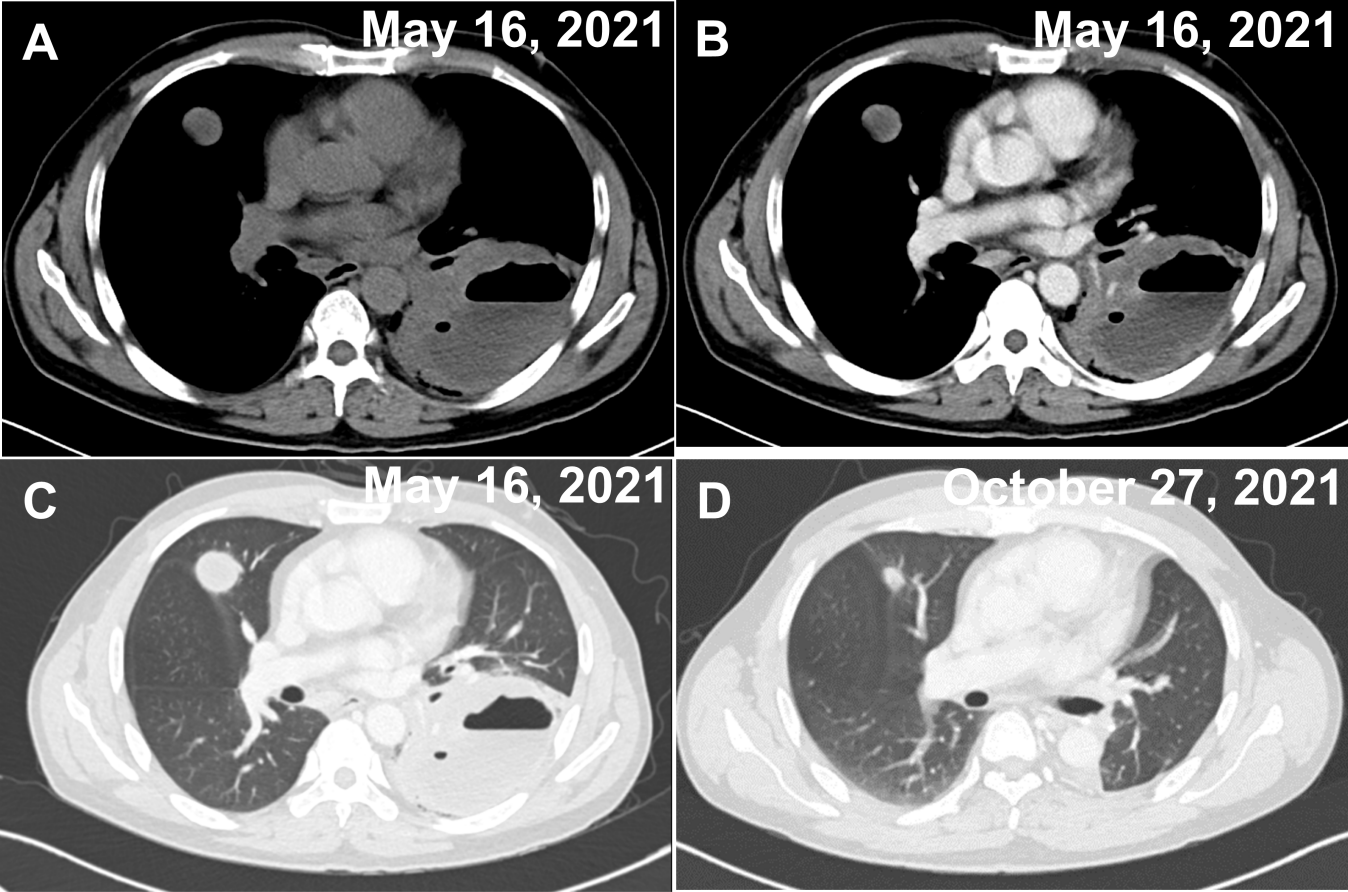
Two CT images of the chest on admission and after treatment. **(A–C)** A cavernous and fluid-flat mass in the lower lobe of the left lung and multiple nodules in the upper lobe of both lungs (the largest nodule is shown in the figure), both of which show mild circumferential enhancement on enhancement scans. **(A)** Unenhanced period. **(B)** Venous phase. **(C)** Pulmonary window. **(D)** Postoperative changes in the left lung, with significantly smaller nodules in the upper lobes of both lungs (largest nodule in the figure) than before.

The patient was a 53-year-old Chinese male with no history of cancer in his immediate family. The patient underwent small intestinal stromal tumor surgery in June 2020 and was treated with oral imatinib after surgery. The patient complained that on April 30, 2021, he had cough and sputum, yellow purulent sputum, with fever, up to 38.8°C, accompanied by chest tightness, no obvious chest pain, and no hemoptysis. After 3 days of infusion in the clinic, he went to a local hospital for treatment. Chest CT showed cavity changes in the left lung, left pleural fluid, and nodules in both lungs. The hospital was given piperacillin + etimicin + levofloxacin anti-infective treatment for 12 days. The cough and sputum were better than before, but there was still fever, and bloodshot sputum appeared. For further diagnosis and treatment, he came to the respiratory department of our hospital on May 14, 2021. Physical examination: thick breath sounds in both lungs, and weak breath sounds in the left lung. Blood routine: C-reaction protein (CRP)158 mg/L, white blood cell (WBC)3.38×10^12/L, red blood cell (RBC)3.36×10^12/L, hemoglobin (HGB)99 g/L, lymphocyte (LYM)0.39×10^9/L, mononuclear cell (MONO)0.74×10^9/L. Hemoglobin A1C (HbA1c) Test: 7.9%. There is no special abnormality in liver and kidney function and electrolyte examination. General bacterial, fungal sputum examination, tuberculosis and other sputum pathogenic examinations were negative. Tumor markers AFP, CEA, CA199, and PSA, were all negative. Electronic bronchoscopy showed mucosal congestion and edema in the basal segment of the left lower lobe, the lumen was narrow, and lung lavage was performed. Chest CT examination was performed on May 16, 2021 (**Figures 1A–C**). On May 17, 2021, a lung biopsy was performed for pathological examination. Symptomatic treatment was given, and a thoracic surgery consultation was recommended to recommend surgery. On June 19, 2021, the left lower lung mass and left lower lung were resected under thoracoscopy, and the left upper lung nodule was wedge-shaped. The surgical specimens were sent for pathological examination. They were then sent to the Department of Pathology, Beijing Friendship Hospital, for genetic testing of T-cell clone analysis using BIOMED-2 PCR protocols. A CHOP chemotherapy regimen (ECOG score 0 points, body surface area 1.52 m², cyclophosphamide 1.1 g1day, pyridoxine 70 mg1day, vinorelbine 40 mg1day, prednisone 100 mg1-5day) was given on August 23, September 16, October 7, October 27, November 18, December 7, 2021, at the Department of Hematology of our hospital, and the CT scan was repeated on October 27, showing that the patient’s condition improved (**Figure 1D**). Radiation therapy was performed in the oncology department of our hospital from January 4 to January 29, 2022. The patient is still under follow-up.

### Pathological result

Pathological diagnosis on left lung biopsy on May 17, 2021, showed lymphomatoid granulomatosis, tending to grade 3.

**Figure 2:**
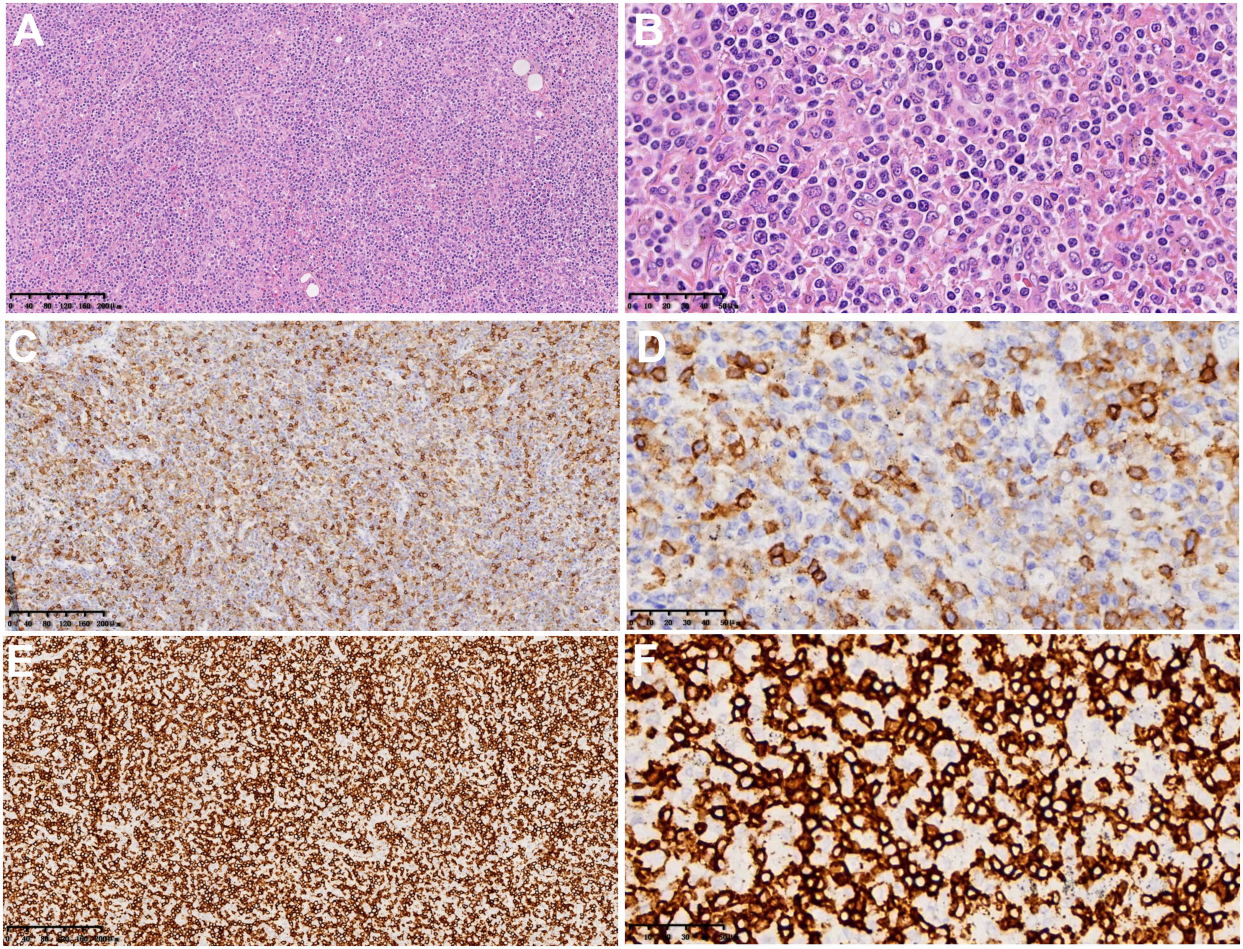
Microscopic findings and immunohistochemistry of surgical pathological specimens: lymphoid cells infiltrated the blood vessel wall in tumor tissue, B lymphocytes scattered in the background of abundant T lymphocytes, and T lymphocytes heteroplasia, with CD8-positive cells as the host. **(A, B)** Hematoxylin-eosin staining (×100 and ×400). **(C, D)** Immunohistochemical results of CD4 (×100 and ×400). **(E, F)** Immunohistochemical results of CD8 (×100 and ×400).

The macroscopic examination of the surgical specimen from June 19, 2021 (**Figure 2**) revealed a 4x1.4x1 cm piece of lung tissue in the left upper lung and a 0.8x0.8x0.7 cm tumor seen in the lung tissue at a distance of 0.6 cm from the lung resection margin. There were many irregular tissues in the left lower lung, totaling 14x12x4 cm, of which a 9x8x4 cm gray–white, gray–yellow slightly hard area could be seen. Microscopic findings: lymphoid cells infiltrated the blood vessel wall in the tumor tissue, scattered B lymphocytes in the rich T lymphocytes background, T lymphocytes heteroplasia, mitotic figures were easy to see, and large necrosis was seen in the center of the tumor. Immunohistochemistry showed tumor tissue Vim(+), CD43, CD2, CD3, CD5, CD7, CD4, CD8 lymphocytes (+); CD56 scattered(+), TiA(+), GrB(+), CD20, CD79a and PAX5 scattered lymphocytes (+); CD10(-), bcl-6(-), Bcl-2(-), Mum(-), C-myc(-), CD21(-), CK23(-), CD30(-), CD15(-), CK(-), S-100(-), CD1a(-), langerin(-), CD68, CD163 histiocytes (+); and Ki-67 dysplastic T lymphocytes were approximately 60% (+). *In situ* hybridization: EBER focal, scattered (+). Pathological diagnosis: (left upper lung nodule) and (left lower lung) T lymphocytes heteroplasia, lymphoma cannot be excluded; genetic testing is recommended to exclude T-cell lymphoma. The 2 lymph nodes of Group 9 submitted for examination showed reactive hyperplasia.

Gene detection results: TCRβvβ+Jβ2 and DB+Jβ1/2 showed monoclonal rearrangement. Pathological diagnosis: pulmonary non-Hodgkin CD8-positive cytotoxic T-cell lymphoma with Epstein–Barr virus infection in some cells.

## Discussion

There are many reports of other primary malignancies in GIST patients, ranging from 4.5% to 42.0% (3–8). Pandurengan et al. (6), in a study of 783 GIST patients, found that other malignancies were more common in GIST patients than in men and older adults. In addition, there were more patients with other malignancies before GIST diagnosis (134 patients) than patients with other malignancies after diagnosis (52 patients). Prostate cancer and breast cancer were the most common malignancies before diagnosis of GIST, and primary malignancies of the lung and kidney were the most common after diagnosis. In the study of 6112 GIST patients, Murphy et al. (7) found that the risk of other malignancies was increased in GIST patients before and after diagnosis. Esophageal cancer and bladder cancer were most common before diagnosis GIST, while ovarian cancer and small bowel cancer were most common after diagnosis GIST. Smith et al. (8) studied 1705 patients with GIST and found that colorectal cancer was the most common cancer to develop within 6 months of diagnosis GIST and that the presence of other malignancies within 6 months of diagnosis GIST was associated with poorer overall survival. In addition, GIST outside the stomach or esophagus and old age were more likely to be complicated with other malignancies.

However, it is unclear whether the presence of other malignancies in GIST patients is an incidental finding, surveillance bias or an advance in detection, or a possible causal relationship (9–13). However, it is unlikely that this is related to imatinib treatment, as other malignancies were also observed prior to the diagnosis of GIST, even at a rate comparable to the rate after diagnosis. At a time when imatinib significantly prolongs survival GIST (14), targeted surveillance and screening of patients diagnosed with GIST may be particularly important.

Primary T-cell lymphoma of the lung are rare, although the risk of GIST in combination with other primary malignancies is high. The diagnostic criteria for PPL (15) are imaging showing pulmonary and bronchial involvement without mediastinal lymph node enlargement; no evidence of lymphoma or lymphocytic leukemia at other sites outside the lungs and bronchi; no previous history of extrathoracic lymphoma diagnosis; and no signs of extrathoracic lymphoma even 3 months after presentation. The imaging presentation of PPL varies and is highly misdiagnosed. It can be classified as nodular or mass, pneumonic or alveolar, cornu, interstitial and mixed, with the nodular mass type being the most common. Most patients with primary pulmonary T-cell lymphoma present with symptoms of fever, cough and dyspnea (16).

In this case, the clinical symptoms were nonspecific. The imaging manifestations were a cavitary mass in the left lower lobe with multiple nodules in the upper lobes of both lungs. No extrapulmonary lymphoma was found during the pre- and postoperative examination of this case, so the diagnosis was made as primary pulmonary lymphoma. It can be easily misdiagnosed and need to be differentiated from a lung abscess, Wegener granulomatosis (WG), lung cancer, and pulmonary tuberculosis.

T-cell lymphoma has a poor prognosis, and the T-cell phenotype is considered an independent and significantly poor prognostic factor (17). An effective treatment strategy for primary pulmonary T-cell lymphoma has not been established, but the use of CHOP chemotherapy regimens has been reported in the literature (18). The patient, in this case, was treated with a CHOP chemotherapy regimen postoperatively with good efficacy.

## Educational message

The main inspirations from this case are the following: First, patients with a history of GIST should be monitored for the possibility of other malignancies. Second, the final diagnosis of primary pulmonary T-cell lymphoma depends on pathologic examination.

## Conclusion

In summary, we report a case of primary pulmonary T-cell lymphoma after operation for small intestinal stromal tumor. The clinical symptoms and imaging examination are not characteristic, so misdiagnosis can easily occur. The final diagnosis still depends on the pathological examination. In addition, patients with a history of GIST should be monitored for other malignancies. The patient, in this case, was treated with a CHOP chemotherapy regimen after surgery with good efficacy and is still being followed up.

## Data availability statement

The original contributions presented in the study are included in the article/supplementary material. Further inquiries can be directed to the corresponding author.

## Ethics statement

The studies involving human participants were reviewed and approved by The Second Affiliated Hospital of Nanchang University Medical Research Ethics Committee. The patients/participants provided their written informed consent to participate in this study.

## Author contributions

ZP and LY wrote the article. YT and ZC collected the clinical information and images of the patients. ZL, AH, and MJ revised the article. FL interpreted the pathological findings. MZ proofread the article. All authors contributed to the article and approved the submitted version.

## Funding

This study was funded by the Key Project of Science and Technology Program of Jiangxi Provincial Department of Education (GJJ200106) and the Applied Research Cultivation Program of Jiangxi Provincial Department of Science and Technology (20212BAG70048).

## Acknowledgments

We thank the Patient for his kind cooperation.

## Conflict of interest

The authors declare that the research was conducted in the absence of any commercial or financial relationships that could be construed as a potential conflict of interest.

## Publisher’s note

All claims expressed in this article are solely those of the authors and do not necessarily represent those of their affiliated organizations, or those of the publisher, the editors and the reviewers. Any product that may be evaluated in this article, or claim that may be made by its manufacturer, is not guaranteed or endorsed by the publisher.
